# Incidence and Prevention of Vaginal Cuff Dehiscence After Laparoscopic and Robotic Hysterectomy in Benign Conditions: An Updated Systematic Review and Meta-Analysis

**DOI:** 10.3390/medicina61040647

**Published:** 2025-04-01

**Authors:** Pier Carlo Zorzato, Riccardo Vizza, Simone Garzon, Mariachiara Bosco, Anna Festi, Alberta Ricci, Irene Porcari, Giacomo Corrado, Rosa Maria Laterza, Stefano Uccella

**Affiliations:** 1 Unit of Obstetrics and Gynecology, Department of Surgery, Dentistry, Pediatrics, and Gynecology, Azienda Ospedaliera Universitaria Integrata Verona, University of Verona, Piazza A. Stefani 1, 37125 Verona, Italy; piercarlo.zorzato@univr.it (P.C.Z.); riccardo.vizza@gmail.com (R.V.); simone.garzon@univr.it (S.G.); mariachiara.bosco@univr.it (M.B.); anna.festi@aovr.veneto.it (A.F.); alberta.ricci@aovr.veneto.it (A.R.); irene.porcari@aovr.veneto.it (I.P.); 2 Dipartimento Scienze della Salute della Donna, del Bambino e di Sanità Pubblica, UOC Ginecologia Oncologica, Fondazione Policlinico Universitario A. Gemelli, IRCCS, 00168 Rome, Italy; giacomo.corrado@policlinicogemelli.it; 3 Division of General Gynecology and Gynecologic Oncology, Department of Obstetrics and Gynecology, Medical University of Vienna, 1090 Vienna, Austria; rosa.laterza@meduniwien.ac.at; 4 Karl Landsteiner Society for Special Gynecology and Obstetrics, 1190 Vienna, Austria

**Keywords:** hysterectomy techniques, postoperative care, surgical outcomes, vaginal cuff closure

## Abstract

*Background and Objectives*: Vaginal cuff dehiscence (VCD) represents a rare but relevant complication that occurs following minimally invasive hysterectomy. With the rising frequency of this procedure, it is crucial to continuously evaluate VCD incidence, risk factors, and prevention strategies. This systematic review and meta-analysis aimed to update the evidence on VCD incidence and to assess the role of various surgical techniques and materials adopted for vaginal cuff closure. *Materials and Methods*: A systematic literature search was conducted in PubMed, Scopus, Web of Science, EMBASE, and the Cochrane Library for studies published up to January 2025. Eligible studies reported VCD rates after laparoscopic or robotic hysterectomy for benign conditions and compared different closure techniques. The primary outcome was the incidence of VCD across closure methods, while secondary outcomes included potential risk factors. A random-effects model estimated pooled VCD rates with 95% confidence intervals (CI), and heterogeneity was assessed using I^2^ tests. *Results*: Twenty-six studies involving 10,039 patients were analyzed. The overall pooled incidence of VCD was 0.7% (95% CI: 0.4–1.1%), with higher estimates in randomized controlled trials (RCTs) (1.4%) compared to non-RCTs (0.5%). Robotic-assisted hysterectomy had a pooled VCD rate of 1.7%, compared to laparoscopic hysterectomy at 0.7%. Although not statistically significant, transvaginal closure showed a higher VCD risk than laparoscopic closure (2.3% vs. 1.16%; OR 0.97, 95% CI, 0.33–2.82; OR 2.53 (95% CI, 1.10–5.82) when considering only RCTs), and barbed sutures showed a lower VCD incidence (0.35%) than conventional sutures (1.52%) (OR 0.37, 95% CI, 0.13–1.02). Smoking was identified as a significant risk factor for VCD, while the impact of early postoperative sexual activity remains inconclusive. *Conclusions*: Laparoscopic closure rather than transvaginal cuff closure and barbed sutures were neither significantly associated with reducing VCD risk. Emphasizing smoking cessation preoperatively is essential for VCD prevention. Future studies should investigate the effects of postoperative sexual activity and refine surgical techniques to minimize VCD risk and improve outcomes.

## 1. Introduction

Vaginal cuff dehiscence (VCD) is a rare but serious postoperative complication following hysterectomy, characterized by partial or complete reopening of the vaginal cuff [1,2]. Although uncommon, VCD can lead to severe consequences, including bowel evisceration, perforation, and sepsis [3].

Minimally invasive hysterectomy has become the preferred surgical approach due to its advantages, such as faster recovery and higher patient satisfaction compared to open surgery [4,5]. However, evidence suggests that VCD occurs more frequently after minimally invasive procedures, such as laparoscopic and robotic-assisted hysterectomy [6]. The estimated incidence of VCD is approximately 1.14% for these techniques, compared to 0.14% for transvaginal hysterectomy and 0.10% for laparotomic hysterectomy [4].

Despite its clinical significance and the high number of hysterectomies performed every year worldwide, there is no clear consensus or standardized guidelines for VCD prevention [7]. While several risk factors have been identified, many cases occur without any apparent predisposing conditions [8,9,10].

This systematic review and meta-analysis synthesizes the latest data to evaluate the incidence of VCD and assess the effectiveness of different surgical techniques and preventive measures in reducing this complication.

## 2. Materials and Methods

### 2.1. Study Design

This systematic review and meta-analysis was designed before the online search, outlining the study population, interventions, comparators, outcome measures, eligibility criteria, and statistical analyses, including subgroup analyses. The study was exempt from institutional review board approval. The methodology followed the Cochrane Handbook for Systematic Reviews of Diagnostic Test Accuracy. It adhered to the Preferred Reporting Items for Systematic Reviews and Meta-Analyses of Diagnostic Test Accuracy (PRISMA-DTA) guidelines [11,12]. The protocol was registered on PROSPERO (CRD 42025642967).

### 2.2. Search Strategy and Eligibility Criteria

A comprehensive literature search was conducted across EMBASE, Scopus, PubMed, Web of Science, and the Cochrane Library from inception until January 2025 by a certified professional librarian (Biblioteca Meneghetti—University of Verona). The search strategy was performed with and without MeSH terms to include non-indexed more recent records and to make the search query replicable in all electronic databases: ((vaginal dehiscence) OR (vaginal cuff dehiscence) OR (vaginal vault dehiscence) OR (vaginal separation) OR (vaginal cuff separation) OR (vaginal vault separation) OR (vaginal breakdown) OR (vaginal cuff breakdown) OR (vaginal vault breakdown)) AND ((laparoscop* OR celioscop* OR Peritoneoscop*) OR (robot*)) AND ((hysterectomy)). Additionally, references from the selected studies were manually reviewed.

Inclusion and exclusion criteria were pre-defined by all contributors, using Population, Intervention, Comparison, and Outcome (PICO) framework. Population: women who underwent conventional laparoscopic or robot-assisted laparoscopic hysterectomy; Intervention: different vaginal cuff closure techniques aimed at preventing dehiscence; Comparison: exploratory strategies compared to standard approaches or alternative closure techniques; Outcome: the primary outcome was the rate of VCD after hysterectomy, while secondary outcomes included potential risk factors such as patients habits or behaviors.

Studies were excluded if they were review articles, case reports with fewer than 30 cases, reports on subtotal or radical hysterectomies, studies involving mixed malignant and benign cases, or lacking relevant outcome data. No language restrictions were applied, and non-English articles were translated [13].

### 2.3. Study Selection and Data Extraction

Two independent reviewers (PCZ, RV) screened titles and abstracts for relevance and assessed the full texts of potentially eligible studies. Disagreements were resolved through discussions with a second reviewer pair (SU, SG). A standardized data extraction form was developed to collect key study details, including study characteristics (first author, publication year, study design, research setting), patient and surgical details (sample size, laparoscopic vs. robotic approach, suture materials, closure techniques), and postoperative outcomes (the incidence of VCD or other complications).

### 2.4. Risk of Bias Assessment

Two reviewers (PCZ and RV) independently assessed the risk of bias using the Cochrane Collaboration method. Bias assessment was evaluated separately for randomized controlled trials (RCTs) and non-randomized studies using the RoB 2 tool for RCTs and the ROBINS-I tool for observational studies [14,15].

### 2.5. Statistical Analysis

The meta-analyses were performed using the random-effect model. The random-effects model was used because the assumption of having a common treatment effect for all included studies required by the fixed-effect model was absent. Because we expected differences in the population characteristics (e.g., age, surgical indication), closure techniques, and surgeon expertise [16], we did not expect a common treatment effect for all included studies but rather that the variation of the impact across studies follows the same distribution.

Proportion meta-analysis was used to estimate the pooled incidence of VCD with 95% confidence interval. The Freeman–Tukey double arcsine transformation was applied to stabilize variance and address zero-cell data issues. Specifically, we used the Freeman–Tukey double arcsine transformation because of the rarity of measured outcomes like VCD. The Freeman–Tukey transformation is particularly suitable because it effectively handles studies with zero-event data, providing more reliable estimates than direct methods. The impact of studies reporting zero events is mitigated by transforming the data, leading to more accurate pooled effect estimates. Wilson Score methods were used to calculate 95% confidence intervals (CIs). Pooled odds ratios (ORs) with 95% CIs were used to investigate factors associated with VCD adopting the inverse variance method with continuity correction in the case of zero-events in one group. Heterogeneity was assessed using the I^2^ statistic, with values < 25% considered low and >75% considered high. All statistical analyses were two-tailed, with a significance threshold of *p* < 0.05. Meta-analyses were conducted using R Statistical Software (v4.1.2; R Core Team 2024).

## 3. Results

### 3.1. Study Selection

**Figure 1:**
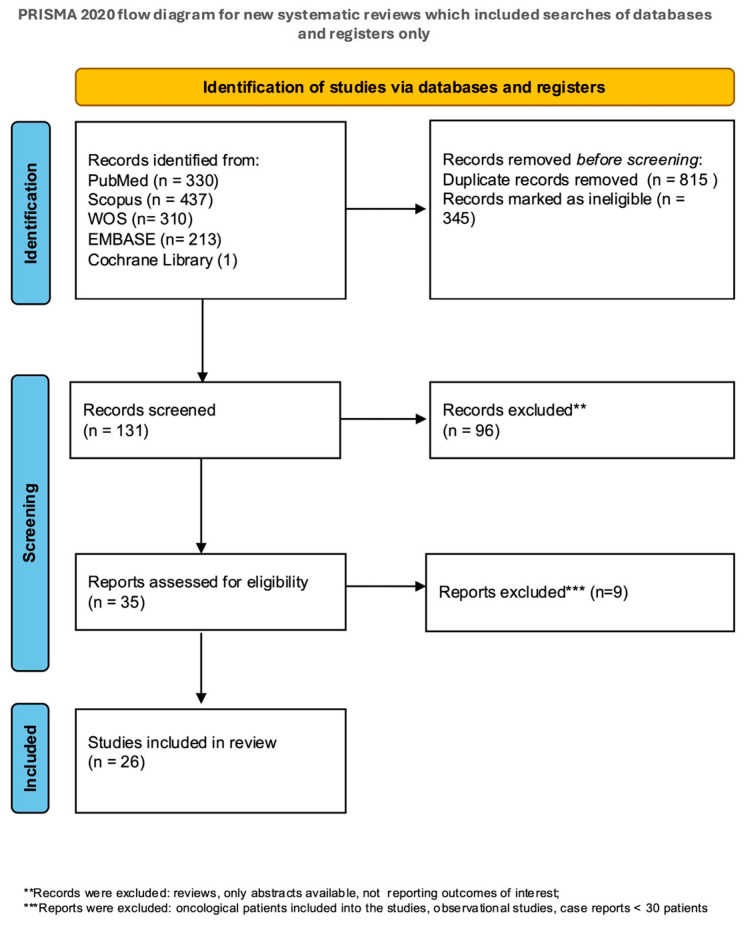
PRISMA flow diagram of reference selection.

The initial literature search retrieved 1068 records, including those identified through cross-referencing. After removing duplicates and non-relevant articles, 131 records underwent title and abstract screening, resulting in 35 potentially relevant articles for full-text review. Ultimately, 26 studies involving 10,039 women met the inclusion criteria and were included in the systematic review and meta-analysis (Table S1). These comprised eight RCTs, 11 retrospective studies, and seven prospective studies [17,18,19,20,21,22,23,24,25,26,27,28,29,30,31,32,33,34,35,36,37,38,39,40,41,42]. The PRISMA Flowchart illustrates the study selection process (Figure 1).

### 3.2. Characteristics of the Included Studies

The included studies evaluating the outcome of vaginal cuff closure after laparoscopic or robotic hysterectomy examined various surgical approaches, types of sutures, and closure methods. In total, the analysis covered 573 robotic-assisted hysterectomies and 9466 laparoscopic hysterectomies. Uccella et al., Hwang et al., López et al., Cong et al., Das et al., and Nawfal et al. conducted comparative analyses of laparoscopic versus vaginal cuff closure [17,21,25,27,28,38]; Nawfal et al. compared laparoscopic to robotic-assisted vaginal closure [21], while Lee et al. and Siedhoff et al. both focused on single-port versus multi-port laparoscopic suturing [23,42].

In addition, 14 studies compared barbed sutures (e.g., V-Loc™, Quill SRS™) with non-barbed sutures (e.g., Vicryl, Poliglactin 910, PDS, Polysorb).

Two studies specifically analyzed different suture materials [Vicryl versus PDS (Cannone et al. [26]), 0-Maxon versus 0-Polysorb (Landeen et al. [39])].

Four studies assessed closure techniques by comparing single-layer vs. double-layer closures. Yildirim et al. and Peter et al. used barbed sutures [34,41], Dojki et al. adopted Vicryl [35], and Jeung et al. compared figure-eight stitches versus continuous running sutures [36].

### 3.3. Risk of Bias Assessment

**Figure 2:**
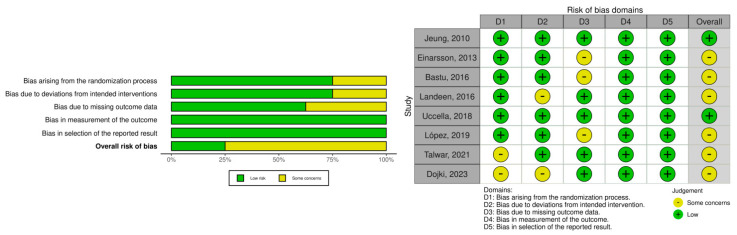
Risk of bias in randomized controlled trials (risk of bias tool for randomized trials) [24,25,32,35,36,38,39,40].

**Figure 3:**
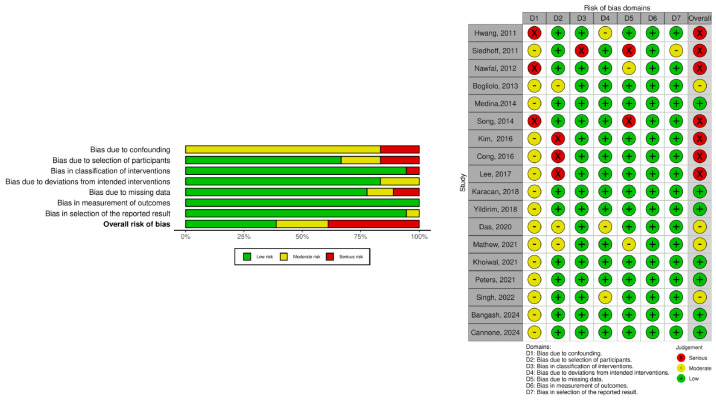
Risk of bias in non-randomized studies (risk of bias in non-randomized studies—of exposures) [17,18,19,20,21,22,23,26,27,28,29,30,31,33,34,37,41].

Bias assessment is summarized in Figure 2 and Figure 3. Despite some methodological concerns, most RCTs demonstrated acceptable quality. However, unclear random sequence generation, allocation concealment, variability in surgical techniques and operator experience, and insufficient follow-up duration may have influenced the reported outcomes. For observational studies, the risk of bias was more pronounced, with many classified as having a moderate or severe risk of bias (Figure 3). Key sources of bias included the lack of randomization and potential confounding due to baseline differences, the retrospective interventions classification and surgeon-dependent procedural choices, the high loss to follow-up and potential underreporting, and, lastly, the selective reporting bias, exacerbated by the absence of study pre-registration and the use of post hoc statistical analyses in certain studies.

### 3.4. Incidence of Vaginal Cuff Dehiscence

Among 10,039 patients who underwent minimally invasive hysterectomy for benign conditions across included studies (retrospective cohorts, prospective cohorts, and randomized controlled trials), 97 cases of VCD were identified, yielding a global pooled incidence of VCD of 0.7% (95% CI: 0.4–1.1%; I^2^ = 59.3%, Chi^2^ = 61.42, df = 25, *p* < 0.01).

Data analysis from seven RCTs revealed a higher VCD rate with a pooled incidence of VCD of 1.4% (95% CI: 0.9–1.9%; I^2^ = 0%, Chi^2^ = 5.4, df = 6, *p* = 0.49). In contrast, non-RCT studies reported a lower rate with a pooled incidence of VCD of 0.5% (95% CI: 0.2–0.9%; I^2^ = 56%, Chi^2^ = 40.5, df = 18, *p* < 0.01). Studies with fewer than 100 patients per arm reported a pooled incidence of VCD of 1.6% (95% CI: 0.2–3.8%; I^2^ = 51%, Chi^2^ = 10.22, df = 5, *p* = 0.07), more extensive studies (≥100 patients per arm) showed a pooled incidence of VCD of 0.7% (95% CI: 0.6–1%; I^2^ = 59%, Chi^2^ = 46.72, df = 19, *p* = 0.01).

Analysis stratified by surgical approach indicated a pooled incidence of VCD of 1.7% (95% CI: 0–5%; I^2^ = 73%, Chi^2^ = 7.3; df = 2, *p* = 0.03) following robotic-assisted hysterectomy versus to a pooled incidence of VCD of 0.7% (95% CI: 0.3–1.2%; I^2^ = 65%, Chi^2^ = 61.99; df = 22, *p* < 0.01) with laparoscopic hysterectomy.

Among 83 cases with detailed documentation, 73 (88%) needed surgical reintervention and repair, whereas in 10 (12%) the conservative management was successful. Additionally, 14 cases of bowel evisceration through the vaginal cuff were reported (Table S1).

### 3.5. Closure Technique

#### Transvaginal vs. Endoscopic Approach to Cuff Closure

**Figure 4:**
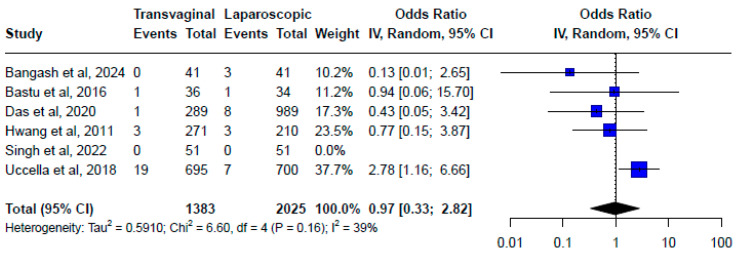
Forest plot. Transvaginal vs. endoscopic approach to cuff closure (OR for vaginal cuff dehiscence of 0.97 (0.33–2.82) with moderate heterogeneity of I^2^ = 39%) [17,28,30,31,38,40].

Two RCTs and four non-randomized studies compared the minimally invasive closure with the transvaginal approach, providing a VCD rate of 1.16% (22/2025) and 2.3% (24/1383), respectively. The estimated odds ratio (OR) for VCD after transvaginal versus minimally invasive approach for the vaginal cuff closure was 0.97 (95% CI, 0.33–2.82; I^2^ = 39%, Chi^2^ = 6.60, df = 4, *p* = 0.16), with no statistically significant difference between the two techniques (Figure 4). However, the meta-analysis limited to the two RCTs provided a pooled OR for VCD after transvaginal (20/731) versus minimally invasive approach (8/734) for the vaginal cuff closure of 2.53 (95% CI, 1.10–5.82; I^2^ = 0%, Chi^2^ = 0.52, df = 1, *p* = 0.47). Notably, five studies found no significant differences in VCD rates between transvaginal and minimally invasive closure techniques [17,28,30,31,40]. Conversely, the larger RCT by Uccella et al. demonstrated a significantly lower VCD rate associated with minimally invasive closure (1% versus 2.7%, *p* = 0.01) [38]. The trial enrolled 1395 patients and was stopped prematurely, due to an exceeding difference (almost three times lower in terms of VCDs) in favor of laparoscopic vs. transvaginal cuff closure.

### 3.6. Use of Barbed Sutures vs. Non-Barbed Sutures

**Figure 5:**
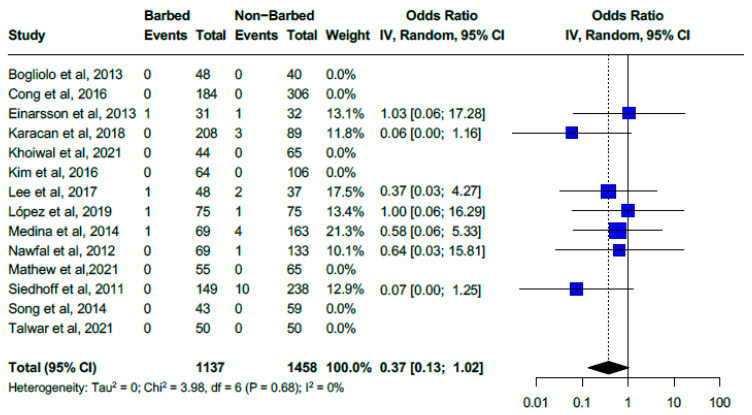
Forest plot. Use of barbed sutures non-barbed sutures for cuff closure (OR for vaginal cuff dehiscence of 0.37 (0.13–1.02) with low heterogeneity of I^2^ = 0%) [18,19,20,21,22,23,24,25,27,29,32,33,37,42].

Among 1137 women who received barbed suture, VCD occurred in 0.35% (4 cases) compared to 1.52% (22/1495) in those with polyglactin or other non-self-anchoring suture types. Barbed suture versus polyglactin or other non-self-anchoring suture reported a non-statistically significant pooled OR for VCD of 0.37 (95% CI, 0.13–1.02). The pooled analysis included fourteen studies (seven retrospective (Figure 5) [18,19,20,21,22,23,42], four prospective studies [18,27,29,37], and three randomized controlled trials (RCTs) [24,25,32]) and heterogeneity was low (I^2^ = 0%, Chi^2^ = 3.98, df = 6, *p* = 0.68). The meta-analysis limited to the three RCTs provided a pooled OR for VCD using barbed suture (2/156) versus polyglactin or other non-self-anchoring suture types (2/157) of 1.02 (95% CI, 0.14–7.38; I^2^ = 0%, Chi^2^ = 0.00, df = 1, *p* = 0.99). Two studies reported a significant reduction in VCD with barbed suture: Siedhoff et al. (387 patients) showed a VCD rate of 4.2% with non-barbed sutures compared to 0% with barbed sutures (*p* = 0.008), while Karacan et al. (297 patients) observed a VCD rate of 3.4% with standard sutures compared to 0% with barbed sutures (*p* = 0.03) [20,23]. The remaining 12 studies found no significant difference in VCD rates between barbed and non-barbed sutures.

### 3.7. Double-Layer vs. Single-Layer Closure

**Figure 6:**
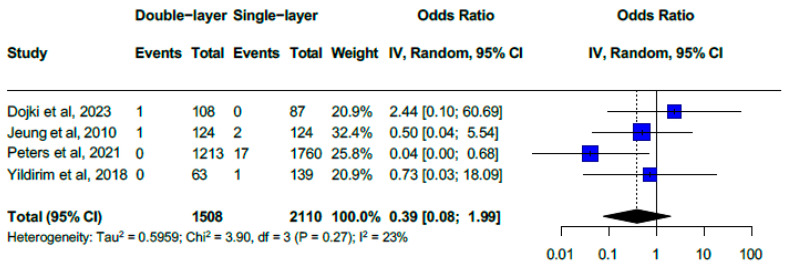
Forest plot. Double-layer vs. single-layer closure for cuff closure (OR for vaginal cuff dehiscence of 0.39 (0.08–1.99) with low heterogeneity of I^2^ = 23%) [34,35,36,41].

Only 2 cases of VCD were reported among 1508 patients who underwent double-layer closure (0.13%, 2/1508) compared to 20 cases out of 2110 patients in the single-layer closure group (0.94%). A pooled analysis of four studies (Figure 6) yielded an overall OR of 0.39 (95% CI, 0.08–1.99) with non-significant low heterogeneity (I^2^ = 23%, Chi^2^ = 3.90, df = 3, *p* = 0.27) [34,35,36,41]. Among individual studies, only Peters et al. reported an OR of 0.04 (95% CI, 0.00 to 0.68). They suggested a benefit of double-layer closure, while the remaining three studies found no significant difference between the two techniques.

### 3.8. Slow-Reabsorption vs. Fast-Reabsorption Sutures

**Figure 7:**
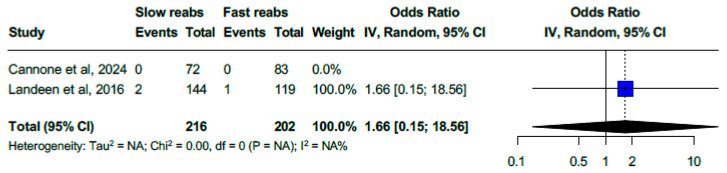
Forest plot. Slow-reabsorption vs. fast-reabsorption sutures for cuff closure (OR for vaginal cuff dehiscence of 1.66 (0.15–18.56); I^2^ = NA) [26,39].

A comparison between slow-reabsorption and fast-reabsorption sutures for vaginal cuff closure showed low event rates in both groups. No cases of VCD were reported in the slow-reabsorption group (0/216), while one (0.5%) case occurred in the fast-reabsorption group (1/202). Additionally, the pooled analysis of the two studies yielded an OR of 1.66 (95% CI, 0.15–18.56) [26,39], further confirming the lack of statistical significance. Due to the limited number of studies, heterogeneity could not be assessed (I^2^ = NA, Chi^2^ = 0.00, df = 0, *p* = NA) (Figure 7).

### 3.9. Smoking as a Risk Factor for VCD

Several studies examined smoking as a potential risk factor for VCD, with varying conclusions. Jeung et al. reported significantly higher postoperative complications in smokers (26.5%) compared to non-smokers (5.1%, *p* < 0.001) [36], identifying smoking, diabetes, and pelvic adhesions as key risk factors. Uccella et al. found smoking to be an independent risk factor for VCD (OR 2.65; 95% CI: 1.09–6.34, *p* = 0.026) [38]. Landen et al. noted that all patients with VCD were smokers, suggesting smoking was the primary risk factor. Das et al. observed that patients who developed VCD were more frequently smokers than those without complications [28,39]. Peter et al. reported that 25.7% of patients were smokers but did not clarify its direct impact on postoperative complications [34].

Other studies provided mixed results: Siedhoff et al. found no significant association between smoking and VCD (*p* = 0.88) [23]. Cannon et al. and Mathew et al. referenced smoking but provided no specific data on its correlation with postoperative complications. Khoiwal et al. acknowledged tobacco in the study but did not include it in statistical models [26,29,33].

### 3.10. Sexual Activity as a Risk Factor for Vaginal Cuff Dehiscence

The impact of postoperative sexual activity on VCD has been investigated, with some studies linking early resumption to higher VCD incidence. Das et al. identified early sexual intercourse as a possible risk factor [28]. Jeung et al. noted that VCD diagnoses often coincided with the first postoperative sexual encounter, suggesting a temporal association [36]. Cannone et al. reported three cases where early sexual activity may have contributed to VCD [26]. Bangash et al. observed that patients with VCD resumed sexual activity earlier than recommended, reinforcing risk hypothesis [31].

Other studies provided limited analysis: Peter et al. documented the timing of sexual resumption but did not clarify its impact on VCD [34]. Medina et al. and Uccella et al. mentioned sexual activity in the context of postoperative recovery but lacked specific data on VCD correlation [37,38]. Hwang et al. and Einarsson et al. did not report findings on sexual activity as a risk factor [17,24].

### 3.11. Time of Onset of Vaginal Cuff Dehiscence

The onset of VCD varies significantly, ranging from a few days to several months postoperatively. Some studies have reported early occurrences. Bangash et al. documented cases within 10 days, Yildirim et al. observed dehiscence as early as 9 days postoperatively, Siedhoff et al. reported a median onset of 46 days (range 15–71 days) [23,31,41], and Karacan et al. found a slightly later median of 56 days (range 14–91 days) [20]. Hwang et al. reported cases occurring within 1 to 2 months post-surgery [17].

Other studies have documented later occurrences of VCD. Jeung et al. observed cases between 49 and 80 days postoperatively, and Einarsson et al. reported a median onset at 66 days (range 42–91 days) [24,36]. Das et al. found a median of 60 days, with cases occurring anywhere from 1 month to 12 months postoperatively [28]. Cannone et al. observed VCD at 61, 73, and 80 days post-surgery. Similarly, Peters et al. described the onset of VCD between 6 and 12 weeks, with one case occurring as late as 5 months postoperatively [34]. Most cases arise in the first 6 to 12 weeks after surgery, though some appear within days, and others may not be evident for several months to a year.

## 4. Discussion

This systematic review and meta-analysis confirms that VCD remains an infrequent but clinically significant complication following minimally invasive hysterectomy, with an incidence of approximately 1–2 every 100 minimally invasive hysterectomies. Despite its low frequency, the consequences are substantial, as 88% of cases require surgical intervention and bowel evisceration occurs in nearly 30% of cases, underscoring the severity of this complication.

In this study, we aimed to identify possible preventive strategies to minimize the risk of VCD occurrence. Regarding the surgical approach, the initial meta-analysis, including all studies, did not observe statistically significant differences when comparing endoscopic versus transvaginal cuff closure. However, when the analysis was limited to RCTs, the minimally invasive approach was significantly associated with a lower rate of VCD versus the transvaginal closure. This is mainly explained by the large RCT by Uccella et al., which provided the most robust evidence on the question, favoring laparoscopic closure (1% vs. 2.7%, *p* = 0.01) [38]. Therefore, although five out of six reports did not observe differences affecting the initial pooled analysis, most are observational studies with a lower quality of evidence than the two RCTs. Therefore, the results of the meta-analysis should be interpreted with caution given the availability of a large RCT, which provides strong evidence in support of the laparoscopic vaginal cuff closure.

Our results may suggest a higher rate of VCD with robotic-assisted hysterectomy (RAH) than conventional laparoscopic approach. Robotic platforms facilitate knot tying compared to conventional laparoscopic surgery. Still, it presents unique challenges in vaginal cuff closure, potentially due to the lack of haptic feedback, which may lead to excessive force application on the suture material or insufficient tension on the thread, increasing the risk of suture damage, slippage and dehiscence [43,44]. Robotic surgeons develop “visual haptic” skills to mitigate this issue, relying on visual cues rather than direct tactile feedback [43,45]. However, caution is mandatory given the observed high heterogeneity and the overlapping 95% CI estimates. VCD rates may also be influenced by other factors not fully accounted for, including case complexity, surgeon experience, and institutional differences in surgical technique, different robotic platforms patient selection. Increased surgical experience with robotic platforms may help mitigate these challenges over time, potentially reducing VCD incidence. Moreover, the integration of next-generation robotic systems with enhanced force feedback may further refine suturing techniques, improving outcomes in robotic hysterectomy [46,47,48,49]. Therefore, current data remain insufficient to achieve conclusions.

Firm conclusions about the effectiveness of barbed sutures in reducing VCD cannot be drawn based on our results. Although a numerical trend favoring barbed sutures over conventional sutures was noted with low heterogeneity, the difference was not statistically significant. Furthermore, no randomized controlled trials with adequate power have been identified. Only three RCTs have directly compared barbed and conventional sutures, all limited by small sample sizes and single-center designs [24,25,32]. Studies by Siedhoff et al. and Karacan et al. reported significant reductions in VCD with barbed sutures. Still, the risk of dehiscence in the control groups was exceedingly high, yet other trials failed to confirm this advantage [20,23]. Among newer technologies, self-retaining sutures have been introduced to optimize tension distribution and eliminate the need for knot tying, potentially improving efficiency and reducing complications. While many systematic reviews and meta-analyses have assessed their safety and effectiveness [50]—five specifically in hysterectomy [1,51,52,53,54]—their impact on VCD remains inconclusive and should be weighed with their theoretical benefits against their added costs and potential risks [55].

Among non-surgery-related risk factors, smoking has been reported to be a significant risk factor for VCD in most studies, possibly for its potential negative impact on tissue vascularization and wound healing [38,56]. Uccella et al. identified smoking as an independent risk factor in their study [38]. At the same time, Landen et al. reported that all patients with VCD in their series were smokers, highlighting its strong correlation with dehiscence and, therefore, the importance of preoperative smoking cessation counseling as part of VCD prevention [39]. While it may seem intuitive to associate smoking with a higher risk of VCD after minimally invasive hysterectomy, the available evidence is quite varied. Studies differ significantly in both their outcomes and in how they relate smoking to other risk factors and various surgical techniques. As a result, the current data do not enable us to draw definitive conclusions about the independent impact of smoking on VCD development.

On the other hand, the role of early postoperative sexual activity remains unclear. While some studies suggest a temporal association between early intercourse and VCD [26,28,36], a lack of quantitative data prevents definitive conclusions [57]. This raises a key research question: What is the optimal duration of postoperative sexual abstinence after hysterectomy to minimize the risk of VCD? Since current abstinence recommendations are based on expert opinion rather than clinical evidence, future research should aim to answer this question by defining the ideal abstinence period and assessing its impact on VCD risk through well-designed clinical studies [58]. Additionally, Enhanced Recovery After Surgery (ERAS) protocols—designed to improve postoperative recovery, minimize complications, and reduce hospital stays—may have potential benefits for VCD prevention [59]. While ERAS has shown positive effects on surgical outcomes, its impact on tissue healing and VCD incidence remains unknown, warranting further research [60,61].

### Strengths and Limitations

This meta-analysis employed a rigorous selection process, improving result reliability and providing updated insights on VCD. However, several limitations must be acknowledged: (1) The limited number of RCTs (only seven included) with only one adequately powered [38]. (2) The risk of allocation bias in non-randomized studies which may have introduced unbalanced variability in closure techniques. (3) The heterogeneity (I^2^ values) observed among studies ranges from low to moderate-high. Concerning VCD incidence, observed moderate-high heterogeneity suggests possible factors affecting reported incidence rate. Notably, only sensitivity analysis limited to RCTs reported low heterogeneity with higher incidence than observational studies. This may suggest the heterogenous risk of missing events with underreporting in observational studies with direct implications for provided results. Additionally, other factors may affect the occurrence and detection of VCD such as differences in patient selection criteria, surgical indications despite all being benign, and the absence of a standardized surgical technique between studies. This is further compounded by operator-dependent factors. Such heterogeneity limits the robustness of pooled analyses and highlights the presence of other factors potentially affecting the occurrence of VCD after total hysterectomy. (4) The limited long-term follow-up data, restricting comprehensive evaluation of VCD occurrence, their recurrence, and the risk of unrecognized delayed onset. (5) The inconsistent data on postoperative sexual activity and timing of symptom onset, complicating a thorough risk factor assessment.

## 5. Conclusions

VCD remains a rare but serious complication following minimally invasive hysterectomy, with significant morbidity. Laparoscopic cuff closure appears superior to transvaginal closure based on a single, adequately powered, randomized controlled trial (Uccella et al., 2018) [38], although the results of the meta-analysis—including both randomized and observational studies—failed to demonstrate a statistically significant difference between the two closure methods. Barbed sutures and double layer technique may provide some benefits; however, large-scale RCTs are needed to validate these findings. The higher VCD rates in RAH highlight the need for improved suturing techniques, surgeon training, advancements in robotic platforms, and the conduction of appropriate RCTs to address all these possible confounders. Addressing modifiable risk factors—particularly smoking cessation and postoperative sexual activity guidelines—may also contribute to reducing VCD risk. Future prospective studies should focus on standardizing best practices, optimizing suture techniques, and exploring the potential role of ERAS protocols in VCD prevention.

## Data Availability

The original contributions presented in this study are included in the article/Supplementary Material. Further inquiries can be directed to the corresponding author.

## Supplementary Materials

The following supporting information can be downloaded at: https://www.mdpi.com/article/10.3390/medicina61040647/s1, Table S1: Characteristics of the studies included.
