# Supplementary material for: Incidence and Prevention of Vaginal Cuff Dehiscence After Laparoscopic and Robotic Hysterectomy in Benign Conditions: An Updated Systematic Review and Meta-Analysis
Source: Medicina (Kaunas). 2025 Apr 1;61(4):647. doi: 10.3390/medicina61040647 (PMC12028423; doi:10.3390/medicina61040647)
Supplement: Supplementary file 1 [file medicina-61-00647-s001.zip › medicina-3547725-supplementary.pdf]

Supplementary Material S1: Search Strategy **Table 1: Characteristics of the studies included**

**Study**	**Study design**	**Type of comparison**	**Patients included, n**	**Rate of dehiscence (%)**	**Cumulative VCD rate**	**Time of presentation of VCD, d (range)**	**Bowel evisceration**	**Re-intervention**
Nawfal et al., 2012	Retrospective	Barbed suture vs polyglactin (RAH)	69 vs 133	0 vs. 0.7	0.04% (1/202)	NR	NR	NR
Karacan et al., 2018	Retrospective	Barbed suture vs polyglactin figure-of-8 (TLH)	208 vs 89	0 vs 3.3	1% (3/297)	14–91	NR	3
Song et al., 2014	Case–control	Barbed suture vs polyglactin (TLH)	59 vs 43	0 vs 0	0 (0/102)	NR	NR	0
Kim et al., 2016	Retrospective	Barbed suture vs 2-layer polyglactin (TLH)	64 vs 106	0 vs 0	0 (0/170)	NR	NR	0
Hwang et al., 2011	Retrospective	Transvaginal suture vs LPS	271 vs 210	1.1 vs 1.4	1.3% (6/481)	NR	NR	6
Siedhoff et al., 2011	Retrospective	Barbed vs. non-barbed suture (TLH)	149 vs 238	0 vs. 4.2	2.5% (10/387)	46 (15-71)	3	8
Bogliolo et al., 2013	Retrospective	Barbed vs polyglactin suture (TLH)	48 vs 40	0 vs 0	0 (0/88)	NR	NR	0
Lee et al., 2017	Retrospective	Barbed vs polyglactin suture (single-port TLH)	48 vs 37	2.7 vs 4.2	3.5% (3/85)	NR	NR	NR
Yildirim et al., 2018	Retrospective	Barbed vs 2-layer polyglactin suture (TLH)	139 vs 63	0.7 vs. 0	0.49% (1/202)	9	NR	0
Medina et al., 2014	Prospective	Double-layer barbed vs X-shaped polyglactin suture (TLH)	69 vs 163	0.4 vs 2.4	2.15% (5/232)	NR	NR	NR
Cong et al., 2016	Prospective	Barbed vs polyglactin suture (TLH)	184 vs 306	0 vs 0	0% (0/490)	NR	NR	NR
Das, 2020	Retrospective	Transvaginal suture vs LPS	289 vs 989	0.34 vs 0.8	0.7 (9/1278)	60 (30-365)	3	7
Mathew, 2021	Prospective	Barbed vs polyglactin sutures (TLH)	55 vs 65	0 vs 0	0 % (0/120)	NR	NR	NR
Khoiwal, 2021	Prospective	Barbed vs polyglactin sutures (TLH)	44 vs 65	0 vs 0	0 % (0/109)	NR	NR	0
Peters, 2021	Retrospective	Double-layer vs single-layer closure of the vault (TLH)	1213 vs 1760	0 vs 0.97	0.57 (17/2973)	Less than 180 days	NR	17
Singh, 2022	Prospective	Transvaginal suture vs LPS	51 vs 51	0 vs 0	0 % (0/102)	NR	NR	NR
Bangash, 2024	Prospective	Transvaginal suture vs LPS	41 vs 41	0 vs 7.3	3.65 (3/82)	Less than 10 days	1	3
Cannone, 2024	Retrospective	Polydioxanone (PDS) vs Vicryl (Poliglactyn 910) (TLH)	72 vs 83	0 vs 0	0 % (0/155)	NR	NR	NR
Jeung et al., 2010	RCT	Double-layer vs single-layer suture (TLH)	124 vs 124	0.8 vs 1.6	1.2% (3/248)	70 (60–80)	1	3
Einarsson et al., 2013	RCT	Barbed vs polyglactin suture (TLH)	32 vs 31	3.1 vs. 3.1	3.17% (2/63)	66 (42–91)	1	2

Bastu et al., 2016	RCT	Transvaginal suture vs LPS	36 vs 34	2.7 vs 2.9	2.85% (2/70)	NR	NR	NR
Landeen et al., 2018	RCT	Single-layer continuous 0-Maxon vs. 3 imbricating figure-of-X polyglactin sutures (RAH)	144 vs 119	1.49 vs. 2.08 vs. 0.8	1.49% (4/263)	NR	NR	NR
Uccella et al., 2018	RCT	Transvaginal suture vs LPS	695 vs 700	2.7 vs. 1	1.86% (26/1395)	NR	5	22
López et al., 2019	RCT	Barbed vs polyglactin suture (TLH)	75 vs 75	1.3 vs 1.14	1.33% (2/150)	NR	NR	2
Talwar, 2021	RCT	Barbed vs. non-barbed sutures (TLH)	50 vs 50	0 vs 0	0 % (0/100)	NR	NR	NR
Dojki, 2023	RCT	Double-layer vs single-layer suture (TLH)	108 vs 87	0.92 vs 0	0.51 (1/195)	NR	NR	0

LAVH = laparoscopically assisted vaginal hysterectomy; LPS = lateral pericardial suture; LRH = laparoscopic radical hysterectomy; NA = not applicable; NR = not reported; RAH = robot-assisted hysterectomy; RCT = randomized controlled trial; TAH = total abdominal hysterectomy; TLH = total laparoscopic hysterectomy; VCD = vaginal cuff dehiscence.
